# PPD skin test positivity and in-hospital mortality across age groups in non-HIV tuberculous meningitis: a retrospective cohort study

**DOI:** 10.3389/fneur.2026.1820517

**Published:** 2026-08-14

**Authors:** Wengao Zeng, Xiangzhi Xiao, Qing Liu, Huashan Zhou, Liu Chen, Yan Ouyang, Zhen Wang, Sufen Chen, Jue Hu, Yanhua Zhou, Qiong Wu

**Affiliations:** 1 Department of Neurology, The Second Hospital of Changsha (West Branch of Changsha Hospital for Maternal & Child Health Care), Changsha, Hunan, China; 2 Department of Pathology, The Second Hospital of Changsha (West Branch of Changsha Hospital for Maternal & Child Health Care), Changsha, Hunan, China; 3 Department of Neurology, The Affiliated Changsha Central Hospital, Hengyang Medical School, University of South China, Changsha, Hunan, China; 4 Department of Otolaryngology, The Second Hospital of Changsha (West Branch of Changsha Hospital for Maternal & Child Health Care), Changsha, Hunan, China; 5 Department of Neurosurgery, The Affiliated Changsha Central Hospital, Hengyang Medical School, University of South China, Changsha, Hunan, China

**Keywords:** age-related heterogeneity, Firth’s logistic regression, in-hospital mortality, PPD skin test, restricted cubic spline, tuberculous meningitis

## Abstract

**Background:**

Purified Protein Derivative (PPD) skin test reactivity reflects cell-mediated immune response to *Mycobacterium tuberculosis*. Evidence regarding the prognostic association of PPD reactivity in TBM, particularly its variation across age groups, remains limited.

**Methods:**

We conducted a single-center retrospective cohort study of consecutive non-HIV TBM patients. Firth’s penalized logistic regression was used to estimate the association between PPD status and in-hospital mortality using fixed covariate sets selected on clinical grounds. Entropy balancing was used as a complementary covariate-balancing analysis. Age-related heterogeneity in the PPD–mortality association was evaluated on multiplicative and additive scales and explored using restricted cubic splines (RCS). Discrimination, calibration, and exploratory decision-curve net benefit were summarized using the area under the receiver operating characteristic curve (AUC), optimism-corrected AUC, Brier score, calibration intercept and slope, and decision curve analysis.

**Results:**

Among 1,574 patients, 187 (11.9%) died during hospitalization. In the fixed clinical model, there was no clear evidence that PPD positivity was associated with mortality in the full cohort (OR 1.30, 95% CI 0.74–2.19) or among adults (OR 0.68, 95% CI 0.31–1.34). Among 211 children, 9 of 25 PPD-positive children and 13 of 186 PPD-negative children died. The age- and diagnostic-category-adjusted pediatric estimate was OR 7.20, 95% CI 2.70–18.94; however, it was based on only 22 pediatric deaths and should be considered hypothesis-generating. The multiplicative interaction OR for adults versus children was 0.08 (95% CI 0.02–0.28), and the pediatric-minus-adult difference in standardized mortality-probability contrasts was 0.321 (95% CI 0.158–0.510). Full-cohort model performance was modest (AUC 0.673; optimism-corrected AUC 0.647; Brier score 0.0994). For the primary pediatric model, the E-value was 4.81 for the point estimate and 2.67 for the lower confidence-limit bound after approximate OR-to-RR conversion.

**Conclusion:**

We found no clear evidence that PPD positivity was associated with in-hospital mortality in the full cohort. The pediatric association was large but imprecise and depended on nine deaths among PPD-positive children; it should be treated as an exploratory signal rather than an established age-dependent prognostic association. Multicenter studies using standardized PPD procedures, quantitative induration and testing-time data, and detailed baseline severity measures are needed to independently replicate the pediatric association.

## Introduction

Tuberculous meningitis (TBM) remains the most devastating form of extrapulmonary tuberculosis, carrying a high risk of death and permanent neurological disability even with prompt antituberculous therapy (1–3). Contemporary cohort studies and meta-analyses report in-hospital mortality ranging from approximately 10 to 30%, with even higher risk among patients presenting with advanced disease severity (2–4). Identifying prognostic markers that reflect host-pathogen interaction and immune status remains an important unmet need in TBM.

The purified protein derivative (PPD) skin test is a classical measure of delayed-type hypersensitivity to *Mycobacterium tuberculosis* antigens and is widely used as an indirect marker of cell-mediated immunity (5, 6). In patients with active tuberculosis, PPD reactivity reflects prior sensitization and a Th1-mediated delayed-type hypersensitivity response. However, in severe forms of tuberculosis, including TBM, cutaneous anergy reflecting impaired cell-mediated immunity has been described, particularly among immunocompromised individuals and young children.

Evidence regarding the prognostic association of PPD reactivity in established TBM remains limited. Most published TBM prognostic studies have focused on clinical stage, hydrocephalus, cerebrospinal fluid parameters, HIV status, and drug resistance, while PPD status has rarely been incorporated into multivariable outcome models (7–9). Evidence regarding whether the PPD–mortality association varies by age is also limited.

Given age-related differences in immune maturation and regulation (10–12), the association between PPD reactivity and mortality may vary across the lifespan. Age-stratified analyses in pediatric TBM may be constrained by limited event counts and therefore require careful consideration of sparse-data bias, residual confounding, and statistical uncertainty.

We therefore examined the association between PPD skin test positivity and in-hospital mortality in non-HIV TBM using Firth models with fixed clinically selected covariate sets and complementary covariate balancing. As a secondary exploratory analysis, we assessed whether adding PPD status to the fixed clinical model improved discrimination, calibration, and decision-curve net benefit.

## Materials and methods

### Study population

**Figure 1:**
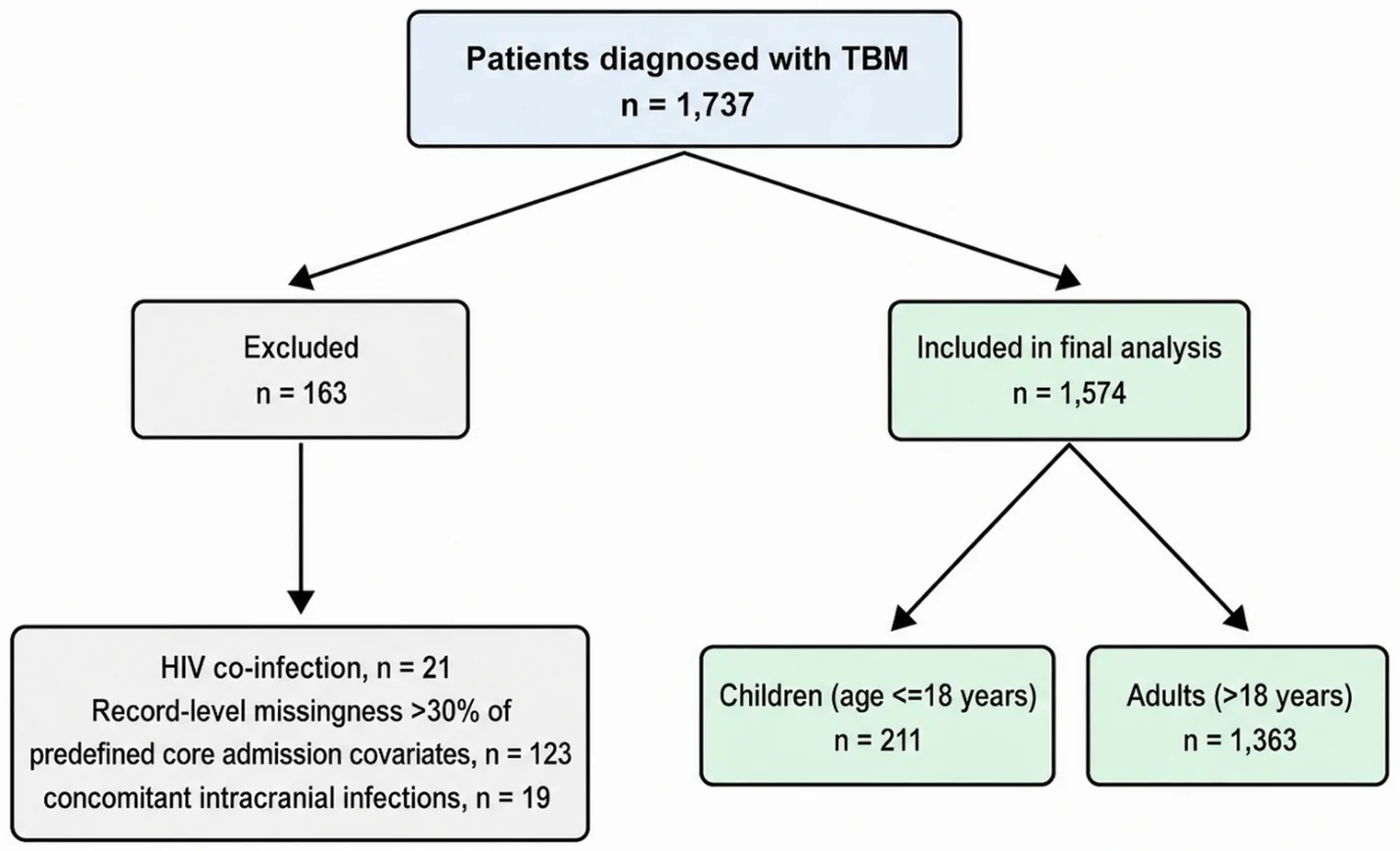
Flow diagram of patient selection. Of 1,737 patients diagnosed with tuberculous meningitis between October 2013 and January 2024, 1,574 non-HIV patients were included in the final analysis after excluding those with HIV co-infection (*n* = 21), record-level missingness exceeding 30% of the predefined core admission covariates (*n* = 123), and concomitant intracranial infections (*n* = 19). The final cohort comprised 211 children (≤18 years) and 1,363 adults (>18 years).

We performed a single-center retrospective observational cohort study of consecutive patients diagnosed with tuberculous meningitis at Changsha Central Hospital between October 1, 2013, and January 1, 2024. The index date (time zero) was the admission date for the index TBM hospitalization. Eligible cases were identified through the hospital electronic medical record system according to predefined inclusion and exclusion criteria (Figure 1).

TBM was diagnosed using internationally accepted consensus criteria integrating clinical manifestations, cerebrospinal fluid (CSF) findings, neuroimaging characteristics, and microbiological evidence (13). Cases were categorized as definite, probable, or possible TBM. Exclusion criteria included: (1) documented HIV co-infection; (2) concomitant intracranial infections, including bacterial or fungal meningitis; and (3) record-level missingness exceeding 30% across the predefined core admission covariates, comprising age, sex, TBM diagnostic category, hydrocephalus, cerebral infarction, immunodeficiency, serum albumin, CSF glucose, hyponatremia, and intracranial pressure. Record-level missingness was calculated as the number of missing core covariates divided by the total number of predefined core covariates within each individual patient record. Patients were categorized as pediatric (≤18 years) or adult (>18 years) based on age at admission. Follow-up was restricted to the index hospitalization. The TST/IGRA component was not used when assigning TBM diagnostic category.

The study was reported in accordance with the Strengthening the Reporting of Observational Studies in Epidemiology (STROBE) guidelines and approved by the institutional ethics committee (Approval No. 20250122) (14). The requirement for informed consent was waived by the Ethics Committee due to the retrospective nature of the study and the use of fully anonymized data, which posed no risk to patient privacy or rights. This waiver is in accordance with the ethical guidelines for biomedical research involving human subjects in China.

### Data collection and variable definitions

Demographic characteristics, clinical manifestations, laboratory findings (blood and CSF), and neuroimaging data were systematically extracted from electronic medical records by two independent investigators. Discrepancies were resolved through joint review and consensus, and data completeness and plausibility were checked before analysis.

Continuous variables were recorded using the earliest available value at hospital admission and before the in-hospital outcome. Descriptive summaries were based on observed values without imputation, and complete-case analysis was used for the variables required by each model. Intracranial pressure was recorded in mmH₂O in the source dataset and converted to cmH₂O by dividing the recorded values by 10.

### Exposure

PPD status was extracted from the electronic medical record as a binary clinical interpretation (positive or negative). Under institutional clinical practice, PPD positivity was generally defined as an induration of ≥10 mm in adults and in children without recognized risk factors, whereas a threshold of ≥5 mm was used in children considered to have recognized risk factors (5, 15). The recorded binary PPD result was used as the exposure.

### Outcome

The primary outcome was in-hospital mortality, defined as death from any cause during the index TBM hospitalization.

### Covariates

Covariates were selected on clinical grounds. The full-cohort and adult models included age, sex, TBM diagnostic category, hydrocephalus, cerebral infarction, immunodeficiency, serum albumin, CSF glucose, hyponatremia, and intracranial pressure. The primary pediatric model included age and diagnostic category to limit overfitting in the sparse pediatric subgroup. A sensitivity model additionally included hydrocephalus and immunodeficiency. Glasgow Coma Scale, level of consciousness, and MRC clinical stage were incompletely recorded, with missingness too extensive for reliable inclusion in the multivariable models.

Hydrocephalus was defined radiologically by ventriculomegaly with an Evans’ index >0.3 (16, 17). Hyponatremia was defined as serum sodium <135 mmol/L within 24 h of admission (18). Cerebral infarction was defined as imaging-confirmed acute or subacute ischemic lesions (19). Immunodeficiency was defined as secondary immunodeficiency, referring to acquired immune dysfunction (excluding HIV), based on laboratory evidence of lymphocyte subset reduction or use of immunosuppressive agents (20). For analysis, immunodeficiency was coded as a binary variable because etiologic subtypes were not consistently recorded. Acute kidney injury was defined according to the kidney disease: improving global outcomes criteria (21).

Brain magnetic resonance imaging (MRI) was performed using a 1.5 T or 3.0 T scanner with T1-weighted, T2-weighted, FLAIR, and contrast-enhanced sequences. Two senior radiologists, blinded to the in-hospital outcome, classified MRI findings into the prespecified categories recorded in the electronic dataset: normal, parenchymal lesions, meningeal enhancement, combined/advanced radiological features, and spinal involvement (Table 1).

**Table 1:** Patient characteristics of TBM patients stratified by outcome (survival vs. non-survival).

Variable	Overall (*N* = 1,574)	Survivors (*N* = 1,387)	Non-survivors (*N* = 187)	*p* value
Demographics				
Age (years), median (IQR)	44.00 (25.00, 59.00)	44.00 (25.00, 59.00)	45.00 (28.00, 62.00)	0.055
Female, n (%)	1,035 (66%)	906 (65%)	129 (69%)	0.400
Comorbidities and clinical features				
Hydrocephalus, n (%)	119 (7.6%)	88 (6.3%)	31 (17%)	**<0.001**
Cerebral hemorrhage, n (%)	16 (1.0%)	11 (0.8%)	5 (2.7%)	**0.033**
Immunodeficiency, n (%)	452 (29%)	374 (27%)	78 (42%)	**<0.001**
Cerebral infarction, n (%)	265 (17%)	228 (16%)	37 (20%)	0.300
Epilepsy, n (%)	86 (5.5%)	61 (4.4%)	25 (13%)	**<0.001**
History of tuberculosis contact, n (%)	318 (20%)	284 (20%)	34 (18%)	0.500
Acute kidney injury, n (%)	412 (26%)	334 (24%)	78 (42%)	**<0.001**
Laboratory findings				
Hyponatremia, n (%)	442 (28%)	367 (26%)	75 (40%)	**<0.001**
Serum creatinine (μmol/L), median (IQR)	48.00 (36.00, 65.00)	47.00 (36.00, 63.00)	54.40 (36.00, 78.00)	**<0.001**
CSF culture positive, n (%)	30 (1.9%)	30 (2.2%)	0 (0%)	**0.041**
CSF white blood cell count (cells/μL), median (IQR)	10.00 (10.00, 60.00)	10.00 (10.00, 51.00)	11.00 (10.00, 100.00)	**0.031**
CSF mononuclear cell proportion, median (IQR)	0.68 (0.50, 0.69)	0.68 (0.50, 0.69)	0.67 (0.50, 0.69)	**0.044**
CSF chloride (mmol/L), median (IQR)	124.20 (115.00, 125.00)	125.00 (116.00, 125.00)	121.00 (113.00, 125.00)	**0.016**
CSF glucose (mmol/L), median (IQR)	2.20 (2.20, 3.21)	2.20 (2.20, 3.21)	2.20 (1.93, 3.21)	0.200
CSF protein (g/L), median (IQR)	0.90 (0.59, 1.37)	0.90 (0.58, 1.32)	0.91 (0.60, 1.84)	**0.013**
CSF ADA (U/L), median (IQR)	2.43 (2.43, 3.00)	2.43 (2.43, 2.80)	2.43 (2.43, 5.00)	**0.038**
CSF *Mycobacterium tuberculosis* DNA detected, n (%)	45 (2.9%)	40 (2.9%)	5 (2.7%)	>0.900
Hemoglobin (g/L), median (IQR)	114.15 (105.00, 126.00)	115.00 (105.00, 126.00)	110.00 (101.70, 119.00)	**<0.001**
Serum albumin (g/L), median (IQR)	35.00 (32.00, 38.50)	35.00 (32.50, 38.80)	34.30 (29.00, 37.00)	**<0.001**
ICP (cmH₂O), median (IQR)	15.0 (12.0, 19.0)	15.0 (12.5, 19.0)	15.0 (12.0, 22.0)	0.200
Drug-resistant tuberculosis, n (%)	98 (6.2%)	83 (6.0%)	15 (8.0%)	0.300
PPD positivity, n (%)	131 (8.3%)	114 (8.2%)	17 (9.1%)	0.700
Diagnostic Tests				
CSF tuberculosis antibody positive, n (%)	195 (12%)	165 (12%)	30 (16%)	0.120
CSF acid-fast bacilli smear positive, n (%)	7 (0.4%)	6 (0.4%)	1 (0.5%)	0.600
Brain MRI findings, n (%)				0.200
Normal	226 (14%)	205 (15%)	21 (11%)	
Parenchymal lesions	1,068 (68%)	939 (68%)	129 (69%)	
Meningeal enhancement	160 (10%)	140 (10%)	20 (11%)	
Combined/advanced radiological features	81 (5.1%)	66 (4.8%)	15 (8.0%)	
Spinal involvement	39 (2.5%)	37 (2.7%)	2 (1.1%)	
TBM diagnostic category, n (%)				0.200
Definite	553 (35%)	493 (36%)	60 (32%)	
Probable	953 (61%)	830 (60%)	123 (66%)	
Possible	68 (4.3%)	64 (4.6%)	4 (2.1%)	

Variables were selected to summarize clinically relevant demographic, clinical, laboratory, imaging, and diagnostic features; variables included in the fixed regression models are also shown. *p* values are descriptive and were not used for covariate selection. Serum albumin was missing for one adult, and cerebral-infarction status was missing for another adult; the remaining variables required for the fixed full-cohort and adult models were complete. PPD-stratified distributions of TBM diagnostic category are provided in Supplementary Table S2. Bold values indicate *p* < 0.05.

Normal: no TBM-related signal abnormality or structural complication.

Parenchymal lesions: infarction, tuberculoma, or encephalitic changes involving brain parenchyma.

Meningeal enhancement: basal, leptomeningeal, linear, or nodular enhancement after contrast administration.

Combined/advanced radiological features: two or more major intracranial complications, including infarction, edema, hemorrhage, or hydrocephalus.

Spinal involvement: spinal cord or subarachnoid abnormalities compatible with TBM extension.

### Statistical analysis

#### Descriptive analysis

Continuous variables were summarized as medians with interquartile ranges, and categorical variables as counts with percentages. Group comparisons used the Wilcoxon rank-sum test or Fisher’s exact test, as appropriate. Diagnostic-category distributions were compared between PPD groups in the full cohort and separately among children and adults (Supplementary Table S2).

#### Model development and variable selection

Associations with in-hospital mortality were estimated using Firth’s penalized logistic regression to reduce small-sample and separation bias (22). Fixed clinically selected models were used for inference to avoid post-selection confidence intervals after data-driven variable selection. The full-cohort and adult models included the covariate set described above. A PPD-only Firth model was fitted as a pediatric univariable benchmark. To limit overfitting in the pediatric subgroup, the primary pediatric model included PPD status, age, and definite versus probable/possible TBM; an additional parsimonious sensitivity model included hydrocephalus and immunodeficiency. Results are reported as odds ratios (ORs) with profile penalized-likelihood 95% confidence intervals (CIs).

**Figure 2:**
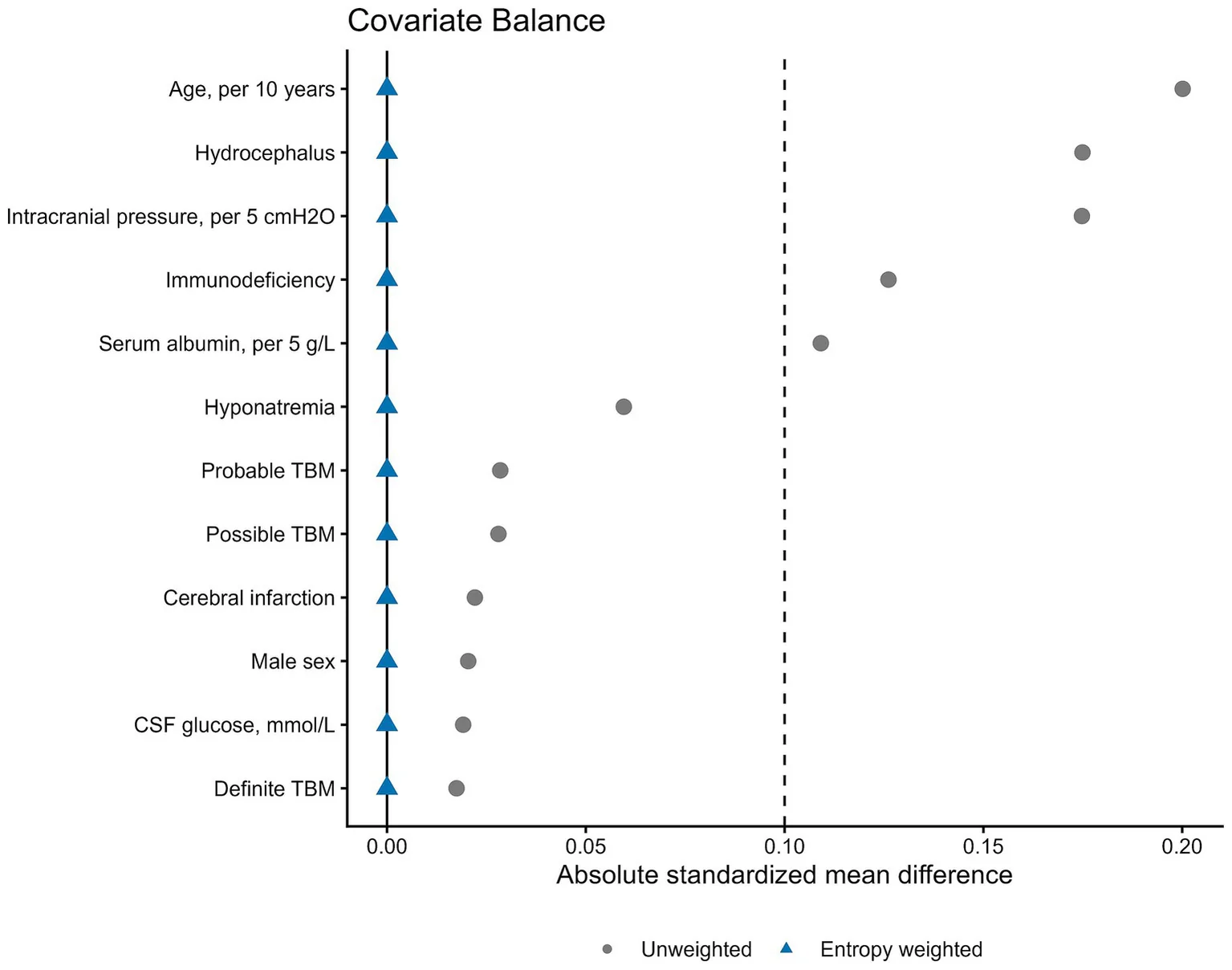
Covariate balance before and after entropy weighting for PPD positivity. Entropy balancing was used to improve covariate balance between PPD-positive and PPD-negative patients. ATE entropy-balancing weights returned by WeightIt were used directly in the weighted outcome analysis. Distinct colors and symbols show unweighted and weighted absolute standardized mean differences; all adjusted values were <0.001.

Entropy balancing for PPD positivity was implemented using the WeightIt package (method = “ebal,” estimand = “ATE”) to improve covariate balance between PPD-positive and PPD-negative patients. The balancing model included age, sex, TBM diagnostic category, hydrocephalus, cerebral infarction, immunodeficiency, serum albumin, CSF glucose, hyponatremia, and ICP, matching the non-exposure covariates in the fixed full-cohort model. Default first-moment constraints were used to balance covariate means; no higher-order moments or covariate-interaction constraints were specified. The ATE weights returned by WeightIt were entered directly into a survey-weighted logistic outcome model fitted with survey:svyglm, with design-based robust standard errors. Balance was assessed using absolute standardized mean differences (Figure 2). A sensitivity analysis truncated weights at the 1st and 99th percentiles.

#### Interaction, nonlinearity, and model performance

Age-group heterogeneity in the PPD–mortality association was assessed using a joint Firth model that included PPD status, the adult-versus-pediatric age-group indicator, their product term, and the covariates in the fixed full-cohort model. Multiplicative heterogeneity was summarized by the product-term OR, representing the ratio of the PPD-associated OR in adults to that in children. The product-term OR was estimated directly from the joint model and was not calculated as the ratio of the separately fitted subgroup ORs.

For the additive-scale analysis, mortality risk was predicted twice for every record within each age group—once with PPD status set to positive and once with it set to negative, while the other covariates were left unchanged—and the predictions were averaged over that age group’s observed covariate distribution. Within each age group, the standardized risk difference was the mean predicted mortality under PPD-positive status minus that under PPD-negative status; additive-scale heterogeneity was then calculated as the pediatric risk difference minus the adult risk difference. Percentile 95% CIs were obtained from 1,000 nonparametric bootstrap resamples.

The age-varying PPD association was explored in the full cohort using rms:lrm with a PPD-by-restricted-cubic-spline interaction for continuous age. Four knots were placed at ages 4, 30, 52, and 74 years (approximately the 5th, 35th, 65th, and 95th percentiles). The model adjusted for sex, TBM diagnostic category, hydrocephalus, cerebral infarction, immunodeficiency, serum albumin, CSF glucose, hyponatremia, and ICP. At each age, the contrast compared PPD-positive with PPD-negative patients at the same age; no single reference age was used. Wald chi-square tests from rms:anova assessed nonlinear and interaction components. Crossings of the point-estimate curve with OR = 1 were identified by linear interpolation and treated as exploratory.

Multicollinearity was assessed with car:vif and reported as generalized variance inflation factors, including GVIF^(1/[2 df]) for multi-degree-of-freedom terms. Discrimination was quantified by the AUC using pROC, and prediction error by the Brier score. Calibration intercepts were estimated from logistic models with the predicted logit as an offset, and calibration slopes from logistic regression of outcome on the predicted logit. AUC optimism was estimated using 1,000 bootstrap resamples as the mean training-minus-test difference and subtracted from the apparent AUC. Decision-curve net benefit was calculated at threshold probabilities of 0.05, 0.10, 0.15, 0.20, and 0.30 for models with and without PPD, with treat-all and treat-none strategies as comparators. For the primary pediatric model, E-values were calculated after converting the OR and its lower 95% CI bound to approximate risk ratios using the square-root approximation for a common outcome.

All analyses were conducted using R software (version 4.5.3). A two-sided *p* < 0.05 was used as the nominal significance threshold. No adjustment was made for multiple testing. *p* values from subgroup, interaction, and spline analyses were considered exploratory and were interpreted with emphasis on effect estimates, uncertainty, and the small pediatric event count. E-values were interpreted as sensitivity measures rather than as hypothesis tests.

#### Treatment

The institutional standard first-line anti-tuberculosis regimen consisted of isoniazid, rifampin, pyrazinamide, and ethambutol. Adjunctive corticosteroids were routinely administered according to institutional practice. Levofloxacin or linezolid was used at the discretion of the treating physician according to clinical judgment, drug-susceptibility results, and disease severity.

## Results

### Patient characteristics

Among 1,574 non-HIV TBM patients, 1,387 survived and 187 (11.9%) died during the index hospitalization. The cohort included 211 children (22 deaths) and 1,363 adults (165 deaths). Documented PPD test results were available for all 1,574 included patients; 131 patients (8.3%) were PPD-positive and 1,443 (91.7%) were PPD-negative. Among children, mortality was 36.0% (9/25) in the PPD-positive group and 7.0% (13/186) in the PPD-negative group. Among adults, mortality was 7.5% (8/106) in the PPD-positive group and 12.5% (157/1,257) in the PPD-negative group. Pediatric characteristics by PPD status are summarized in Supplementary Table S1. Among PPD-positive and PPD-negative patients, the distributions of definite, probable, and possible TBM were similar in the full cohort (Fisher *p* = 0.973), children (*p* = 1.000), and adults (*p* = 0.876; Supplementary Table S2).

Among children, the median age was 10.0 years (IQR 2.2–14.8) in those who died and 8.0 years (IQR 3.0–15.0) in survivors (*p* = 0.953). Immunodeficiency was present in 3/22 (13.6%) children who died and 18/189 (9.5%) survivors (*p* = 0.466; Supplementary Table S3).

**Figure 3:**
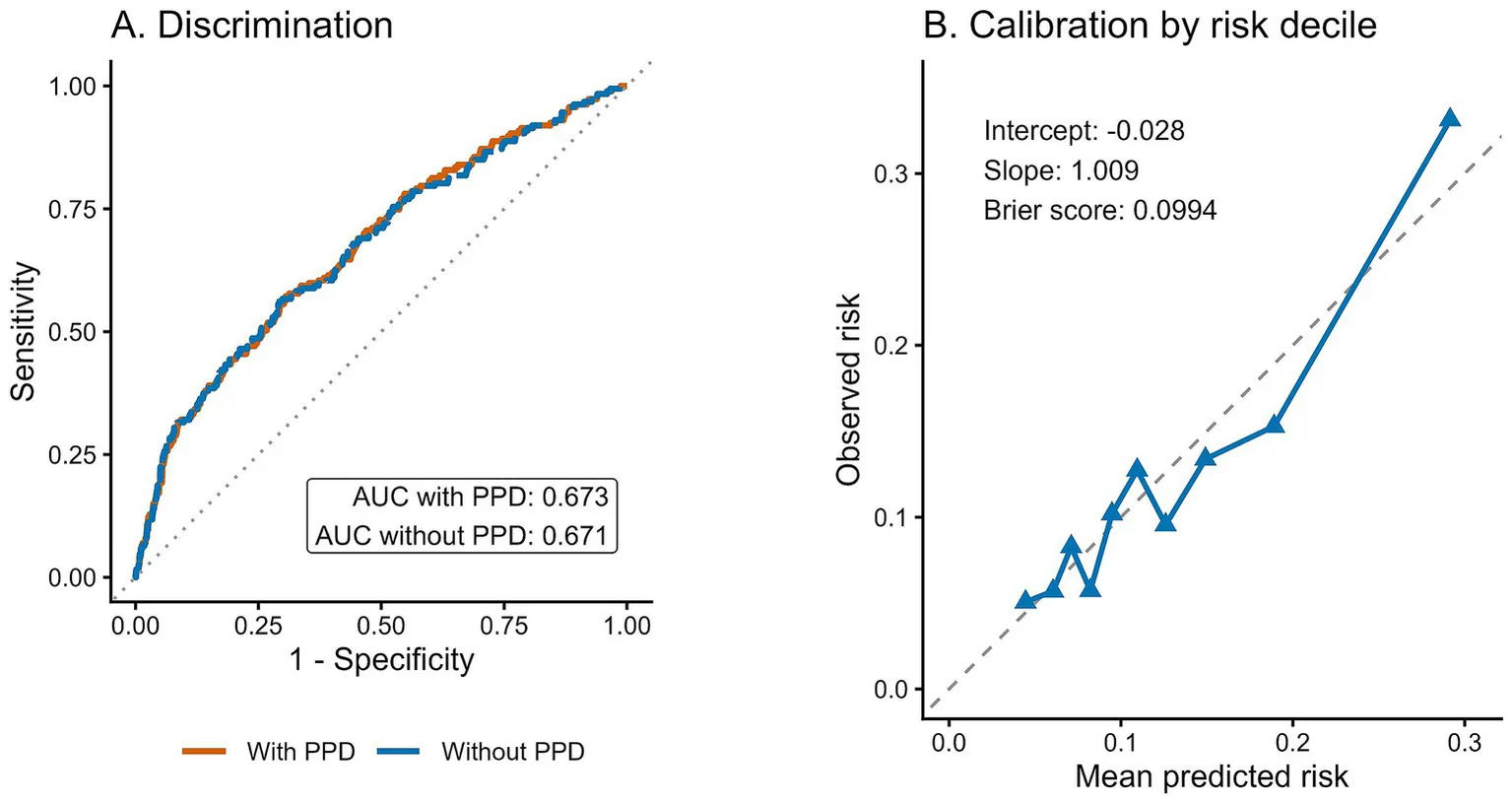
Discrimination and apparent calibration of the fixed clinical model for in-hospital mortality. **(A)** Compares the receiver operating characteristic curves of the models with and without PPD. **(B)** Shows apparent calibration by risk decile for the model with PPD. The model with PPD yielded an apparent AUC of 0.673, a 1,000-bootstrap optimism-corrected AUC of 0.647, a Brier score of 0.0994, a calibration intercept of −0.028, and a calibration slope of 1.009. The corresponding model without PPD yielded an apparent AUC of 0.671 and an optimism-corrected AUC of 0.651. The apparent AUC was 0.002 higher with PPD, whereas the optimism-corrected AUC was 0.004 lower.

**Figure 4:**
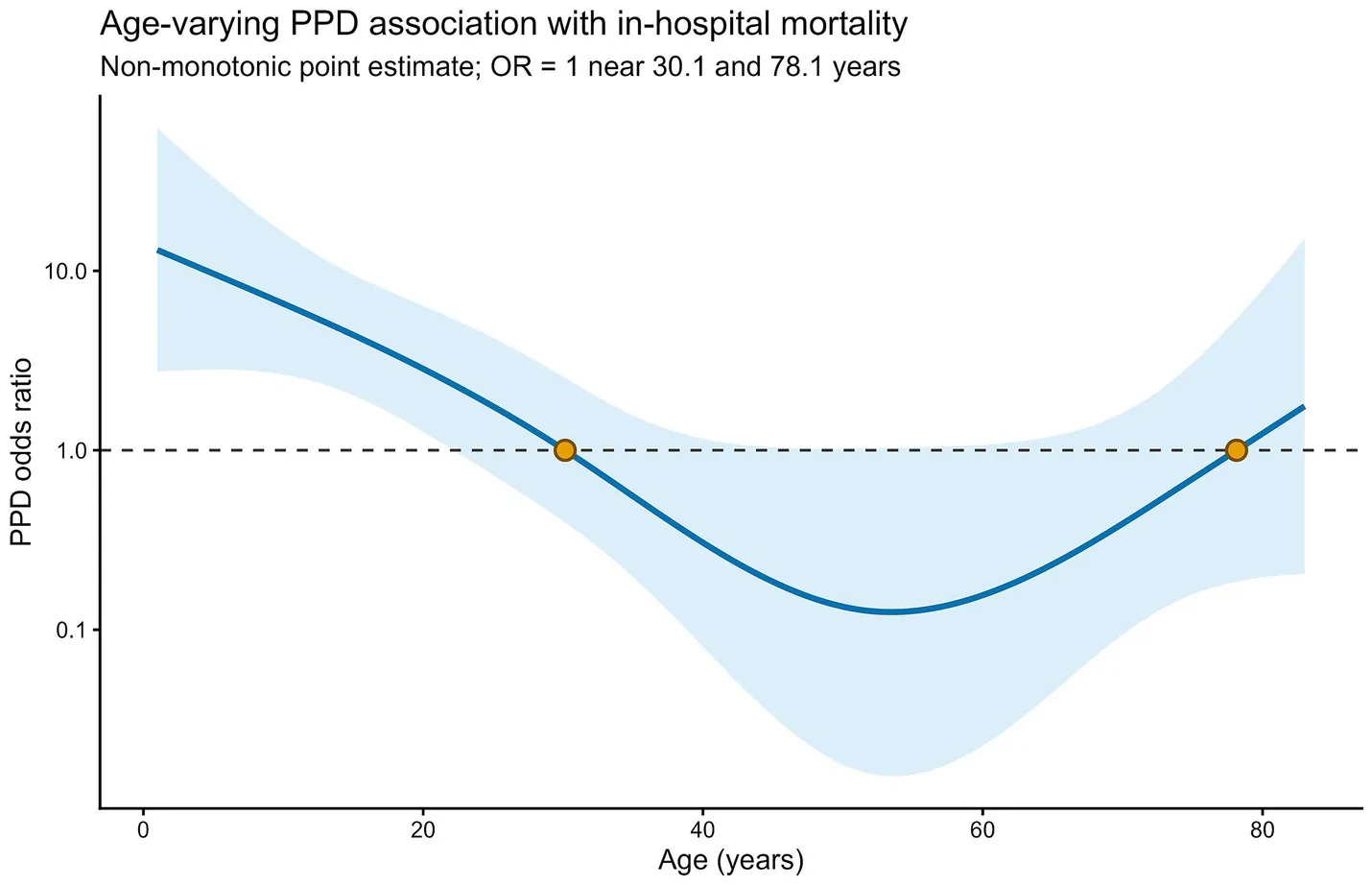
Age-varying odds ratio for PPD-positive versus PPD-negative status from a restricted cubic spline interaction model. The solid curve shows the estimated odds ratio and the shaded band the 95% CI; the dashed horizontal line denotes *OR* = 1. The overall age-by-PPD spline interaction had *p* = 0.002, and the nonlinear interaction component had *p* = 0.034. The point-estimate curve was non-monotonic and crossed *OR* = 1 at approximately 30.1 and 78.1 years. The 95% confidence band was wide near both crossings.

**Figure 5:**
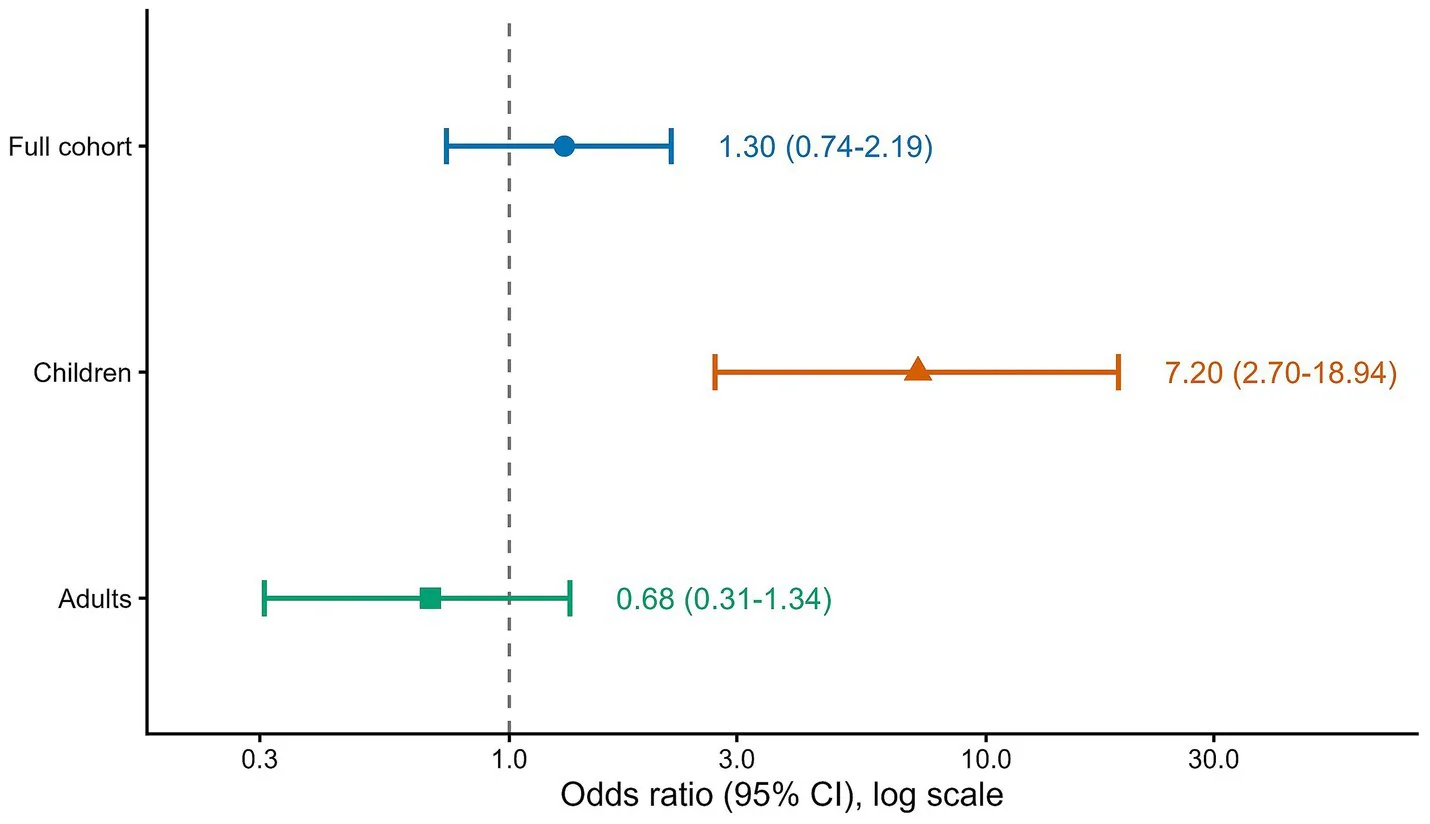
PPD estimates from the fixed full-cohort, pediatric, and adult models. Full-cohort and adult estimates use the fixed clinical model; the pediatric estimate is adjusted for age and diagnostic category. Points show ORs and lines show 95% CIs. The pediatric model was based on 211 children and 22 deaths. The full-cohort and adult models included age, sex, diagnostic category, hydrocephalus, cerebral infarction, immunodeficiency, serum albumin, CSF glucose, hyponatremia, and intracranial pressure. The pediatric model included age and diagnostic category. Complete model coefficients are presented in Table 2.

The fixed full-cohort and adult analyses included 1,572 and 1,361 complete cases, respectively; two different adults were excluded, one because serum albumin was missing and one because cerebral-infarction status was missing. The full-cohort estimate was OR 1.30 (95% CI 0.74–2.19; *p* = 0.346), and the adult estimate was OR 0.68 (95% CI 0.31–1.34; *p* = 0.284); both 95% CIs included 1. Among children, a directly specified univariable Firth model yielded OR 7.40 (95% CI 2.76–19.62; *p* < 0.001). After adjustment for age and diagnostic category, the primary pediatric estimate was OR 7.20 (95% CI 2.70–18.94; *p* < 0.001). A parsimonious sensitivity model including age, diagnostic category, hydrocephalus, and immunodeficiency yielded OR 9.42 (95% CI 3.33–27.40; *p* < 0.001). The multiplicative product-term OR comparing adults with children was 0.08 (95% CI 0.02–0.28; *p* < 0.001); the product-term estimate was obtained directly from the joint interaction model. On the additive scale, the standardized mortality-probability contrast between PPD-positive and PPD-negative records was 0.321 greater in children than in adults (95% CI 0.158–0.510). The pediatric analyses were based on 22 deaths, including nine among PPD-positive children (Table 2). Entropy balancing achieved absolute standardized mean differences <0.001; entropy-balancing weights ranged from 0.398 to 2.479. The weighted outcome OR was 1.06 (95% CI 0.59–1.90; *p* = 0.846), and the estimate after truncating the weights at the 1st and 99th percentiles was 1.00 (95% CI 0.58–1.74; *p* = 0.991; truncated range, 0.648–1.428; Supplementary Table S6). The maximum adjusted generalized variance inflation factor was 1.79. The full-cohort model with PPD had an AUC of 0.673, optimism-corrected AUC of 0.647, Brier score of 0.0994, calibration intercept of −0.028, and calibration slope of 1.009; the corresponding model without PPD had an AUC of 0.671 and optimism-corrected AUC of 0.651 (apparent difference in AUC, 0.002; Supplementary Table S4). The apparent AUC was 0.002 higher with PPD, whereas the optimism-corrected AUC was 0.004 lower (Figure 3). Across threshold probabilities of 0.05–0.30, the absolute difference in net benefit between models with and without PPD did not exceed 0.0010; detailed values are reported in Supplementary Table S5 and Supplementary Figure S1. In the restricted cubic spline model, the Wald chi-square statistic was 14.34 (df = 3; *p* = 0.002) for the overall age-by-PPD interaction and 6.77 (df = 2; *p* = 0.034) for the nonlinear interaction component. The point-estimate curve was non-monotonic and crossed the null value of OR = 1 at approximately 30.1 and 78.1 years of age; the 95% confidence band was wide in the vicinity of both crossing points (Figure 4). The PPD-associated odds ratio estimates for the full cohort, the pediatric subgroup, and the adult subgroup are summarized in Figure 5.

**Table 2:** Firth penalized logistic regression models for in-hospital mortality.

Model/variable	OR (95% CI)	*p* value
Full cohort		
PPD positive	1.30 (0.74–2.19)	0.346
Age, per 10 years	1.00 (0.92–1.09)	0.950
Male sex	0.87 (0.62–1.21)	0.406
Probable TBM vs. definite	1.18 (0.83–1.67)	0.361
Possible TBM vs. definite	0.46 (0.14–1.17)	0.107
Hydrocephalus	3.15 (1.95–4.99)	<0.001
Cerebral infarction	0.90 (0.58–1.35)	0.603
Immunodeficiency	1.76 (1.24–2.50)	0.002
Serum albumin, per 5 g/L	0.76 (0.66–0.88)	<0.001
CSF glucose, per mmol/L	0.95 (0.83–1.08)	0.436
Hyponatremia	1.40 (0.99–1.97)	0.060
Intracranial pressure, per 5 cmH₂O	1.07 (0.97–1.18)	0.155
Children: univariable benchmark		
PPD positive	7.40 (2.76–19.62)	<0.001
Children: primary		
PPD positive	7.20 (2.70–18.94)	<0.001
Age, per 5 years	1.00 (0.69–1.44)	0.995
Probable/possible TBM vs. definite	1.41 (0.57–3.56)	0.454
Children: sensitivity		
PPD positive	9.42 (3.33–27.40)	<0.001
Age, per 5 years	1.03 (0.70–1.51)	0.881
Probable/possible TBM vs. definite	1.48 (0.59–3.79)	0.403
Hydrocephalus	5.08 (1.59–15.88)	0.007
Immunodeficiency	1.69 (0.38–5.94)	0.456
Adults		
PPD positive	0.68 (0.31–1.34)	0.284
Age, per 10 years	1.00 (0.90–1.12)	0.934
Male sex	0.83 (0.57–1.19)	0.314
Probable TBM vs. definite	1.13 (0.78–1.65)	0.534
Possible TBM vs. definite	0.45 (0.14–1.16)	0.103
Hydrocephalus	3.08 (1.82–5.10)	<0.001
Cerebral infarction	0.92 (0.59–1.40)	0.691
Immunodeficiency	1.77 (1.23–2.55)	0.002
Serum albumin, per 5 g/L	0.77 (0.66–0.91)	0.001
CSF glucose, per mmol/L	0.95 (0.82–1.08)	0.426
Hyponatremia	1.35 (0.93–1.94)	0.117
Intracranial pressure, per 5 cmH₂O	1.06 (0.96–1.18)	0.261

ORs are shown with profile penalized-likelihood 95% CIs. The full-cohort model included 1,572 complete cases and 187 deaths; the adult model included 1,361 complete cases and 165 deaths. Two adults were excluded because one lacked serum albumin and one lacked cerebral-infarction status. Both models use the fixed clinical covariate set. The directly specified pediatric univariable benchmark included all 211 children and 22 deaths and contained PPD status only. The primary pediatric model included all 211 children and 22 deaths and adjusted for age and definite versus probable/possible TBM; the parsimonious sensitivity model additionally included hydrocephalus and immunodeficiency. The pediatric models were based on 211 children and 22 deaths. All fitted terms from the fixed models are displayed.

After approximate conversion of the pediatric OR to a risk ratio, the E-value was 4.81 for the point estimate and 2.67 for the lower confidence-limit bound.

## Discussion

In this retrospective cohort, the full-cohort estimate was compatible with no association but remained imprecise after adjustment for fixed clinically selected covariates (OR 1.30, 95% CI 0.74–2.19). A large pediatric association persisted after limited adjustment (OR 7.20, 95% CI 2.70–18.94), whereas the adult CI included the null (OR 0.68, 95% CI 0.31–1.34). Interaction estimates suggested age-related heterogeneity on both multiplicative and additive scales, although they were driven by sparse pediatric events: only 22 children died, including nine PPD-positive children. The pediatric association should therefore be interpreted as hypothesis-generating rather than as an established prognostic association.

TBM diagnostic category was similarly distributed between PPD groups in the full cohort and within pediatric and adult strata. Nevertheless, most cases were probable or possible rather than definite TBM. Microbiological confirmation was limited, and no CSF culture-positive case was recorded among non-survivors. Diagnostic-category balance therefore cannot exclude diagnostic misclassification or residual confounding by incompletely recorded neurological-severity measures, including level of consciousness, Glasgow Coma Scale, and MRC clinical stage (9, 23–26).

The pediatric estimate is statistically fragile. Nine of 25 PPD-positive children died, compared with 13 of 186 PPD-negative children. Although Firth penalization reduces small-sample and separation bias, it does not create information that is absent from sparse cells. The wide confidence intervals across the pediatric models indicate substantial uncertainty, and a small number of outcome reclassifications could materially alter the estimate. The pediatric finding should therefore be considered a signal for external investigation.

Median age was 10.0 years among pediatric deaths and 8.0 years among pediatric survivors (*p* = 0.953). The immunodeficiency estimate was imprecise and did not provide clear evidence of an association with pediatric mortality. Measured characteristics were broadly overlapping between PPD-positive and PPD-negative children, but estimates were imprecise because the PPD-positive subgroup included only 25 patients, and clinically meaningful imbalances cannot be excluded. However, these descriptive comparisons do not address incompletely recorded neurological severity, diagnostic delay, nutritional status, or host susceptibility (27–30).

Age-related immune maturation is one possible hypothesis for the observed heterogeneity (10–12, 28, 31–35); however, the present study did not measure the immune features required to evaluate this mechanism, including cytokine profiles, immunophenotypes, PPD induration diameter, or longitudinal immune responses. The RCS point-estimate curve was non-monotonic and crossed OR = 1 at approximately 30.1 and 78.1 years, but the confidence intervals were wide. These crossings are model-dependent exploratory observations rather than biological or clinical age thresholds.

In adults, the adjusted estimate was compatible with both a modest inverse association and no association. PPD reactivity should not be interpreted as protective. The fixed clinical model showed modest discrimination (AUC 0.673; optimism-corrected AUC 0.647). The corresponding model without PPD had an AUC of 0.671 and optimism-corrected AUC of 0.651. The apparent AUC was 0.002 higher with PPD, whereas the optimism-corrected AUC was 0.004 lower (Figure 3). Apparent in-sample calibration was close to ideal; however, the optimism-corrected AUC and decision-curve net-benefit estimates did not materially differ between models with and without PPD. PPD status may warrant further investigation as a host-response marker, but the present results do not support its use as a stand-alone prognostic predictor.

Strengths of this study include the large overall cohort, explicit diagnostic-category comparisons, Firth penalization, fixed clinically selected covariate sets, complementary covariate balancing, analyses of age-group heterogeneity on multiplicative and additive scales, and reporting of calibration and decision-curve metrics.

The principal limitation is residual confounding. Glasgow Coma Scale, level of consciousness, and MRC clinical stage were incompletely recorded and therefore could not be included in multivariable adjustment, although they are established predictors of TBM mortality. The analysis also lacked time from symptom onset to treatment, detailed nutritional measures, inflammatory biomarker profiles, and timing of death (36). Fixed clinically selected models reduce dependence on data-driven variable selection but cannot eliminate bias from these incompletely measured or unavailable factors. After approximate OR-to-RR conversion, the E-value was 4.81 for the pediatric point estimate and 2.67 for the lower confidence-limit bound. These values indicate that unmeasured confounding would need to be associated with both PPD positivity and mortality by risk ratios of approximately 4.81 to explain the point estimate, or 2.67 to move the confidence interval to the null, conditional on measured covariates. However, confounding of clinically important magnitude remains plausible because direct neurological-severity measures were incompletely recorded and could not be included; therefore, the E-value does not establish robustness.

Other limitations include the single-center retrospective design; only 22 pediatric deaths; predominance of probable or possible TBM and limited microbiological confirmation; a binary immunodeficiency variable without consistently recorded etiologic subtype information; absence of mortality timing and post-discharge follow-up; and no independent external replication.

The timing of PPD placement and reading relative to admission and death was unavailable, so measurement-timing bias cannot be excluded. The dataset retained only the binary clinical interpretation and did not include exact induration diameter, standardized testing time, or IGRA results (37–39). Because a lower positivity threshold was applied to selected high-risk children, differential exposure classification across age groups may have contributed to the observed heterogeneity. Exact induration measurements were unavailable for a sensitivity analysis using a uniform threshold. Consequently, the intensity of PPD reactivity could not be evaluated, and false-negative classification related to anergy remains possible.

## Conclusion

We found no clear evidence that PPD positivity was associated with in-hospital mortality in the full cohort. The pediatric association was large but imprecise, whereas the adult CI included the null. Because the pediatric result depended on nine deaths among PPD-positive children and important severity measures were incompletely recorded and could not be included in multivariable adjustment, it should not be considered definitive evidence of an age-dependent prognostic association. Prospective multicenter studies should standardize PPD administration, reading, and timing; retain quantitative induration measurements and IGRA results; systematically record GCS, MRC stage, and treatment delay; and accrue sufficient pediatric events for independent replication.

## Data Availability

The original contributions presented in the study are included in the article/Supplementary material, further inquiries can be directed to the corresponding author.

## References

[1] Evans EE Avaliani T Gujabidze M Bakuradze T Kipiani M Sabanadze S . Long term outcomes of patients with tuberculous meningitis: the impact of drug resistance. PLoS One. (2022) 17:e0270201. doi: 10.1371/journal.pone.0270201, 35749509 PMC9232145

[2] du Preez K Jenkins HE Donald PR Solomons RS Graham SM Schaaf HS . Tuberculous meningitis in children: a forgotten public health emergency. Front Neurol. (2022) 13:751133. doi: 10.3389/fneur.2022.751133, 35370901 PMC8970690

[3] Stadelman AM Ellis J Samuels THA Mutengesa E Dobbin J. Treatment outcomes in adult tuberculous meningitis: a systematic review and meta-analysis. Open Forum Infect Dis. (2020) 7:ofaa257. doi: 10.1093/ofid/ofaa257 32818138 PMC7423296

[4] du Preez K Jenkins HE Martinez L Chiang SS Dlamini SS Dolynska M . Global burden of tuberculous meningitis in children aged 0-14 years in 2019: a mathematical modelling study. Lancet Glob Health. (2025) 13:e59–68. doi: 10.1016/S2214-109X(24)00383-8, 39706662 PMC11729397

[5] World Health Organization. WHO Operational Handbook on tuberculosis. Module 5: Management of tuberculosis in children and Adolescents. Geneva: World Health Organization. (2022). 35404556

[6] Pahal P Pollard EJ Sharma S. "PPD Skin Test". In: StatPearls. Treasure Island (FL): StatPearls Publishing (2025) 32310497

[7] Donovan J Cresswell FV Tucker EW Davis AG Rohlwink UK Huynh J . A clinical practice guideline for tuberculous meningitis. Lancet Infect Dis. (2026) 26:e96–e111. doi: 10.1016/S1473-3099(25)00364-0, 40840485 PMC12419961

[8] Wang M-G Luo L Zhang Y Liu X Liu L He JQ. Treatment outcomes of tuberculous meningitis in adults: a systematic review and meta-analysis. BMC Pulm Med. (2019) 19:200. doi: 10.1186/s12890-019-0966-8, 31694599 PMC6833188

[9] Gu J Xiao H Wu F Ge Y. Prognostic factors of tuberculous meningitis: a single-center study. Int J Clin Exp Med. (2015) 8:4487–93. 26064373 PMC4443207

[10] Basu Roy R Whittaker E Seddon JA Kampmann B. Tuberculosis susceptibility and protection in children. Lancet Infect Dis. (2019) 19:e96–e108. doi: 10.1016/S1473-3099(18)30157-9, 30322790 PMC6464092

[11] Simon AK Hollander GA McMichael A. Evolution of the immune system in humans from infancy to old age. Proc Biol Sci. (2015) 282:20143085. doi: 10.1098/rspb.2014.3085, 26702035 PMC4707740

[12] Maddux AB Douglas IS. Is the developmentally immature immune response in paediatric sepsis a recapitulation of immune tolerance? Immunology. (2015) 145:1–10. doi: 10.1111/imm.12454, 25691226 PMC4405319

[13] Marais S Thwaites G Schoeman JF Török ME Misra UK Prasad K . Tuberculous meningitis: a uniform case definition for use in clinical research. Lancet Infect Dis. (2010) 10:803–12. doi: 10.1016/S1473-3099(10)70138-9, 20822958

[14] von Elm E Altman DG Egger M Pocock SJ Gøtzsche PC Vandenbroucke JP. The strengthening the reporting of observational studies in epidemiology (STROBE) statement: guidelines for reporting observational studies. J Clin Epidemiol. (2008) 61:344–9. doi: 10.1016/j.jclinepi.2007.11.008, 18313558

[15] Singhal T. The new WHO consolidated guidelines for Management of Tuberculosis in children and adolescents: an appraisal. Indian J Pediatr. (2022) 89:743–5. doi: 10.1007/s12098-022-04280-3, 35612685

[16] Yin R Wen J Wei J. Progression in neuroimaging of normal pressure hydrocephalus. Front Neurol. (2021) 12:700269. doi: 10.3389/fneur.2021.700269 34867705 PMC8636440

[17] Farb R Rovira A. "Hydrocephalus and CSF disorders". In: Hodler J Kubik-Huch RA Schulthess GK, editors. Diseases of the Brain, Head and Neck, Spine 2020–2023: Diagnostic Imaging. Cham (CH): Springer (2020)

[18] Heinrich S Wagner A Gross P. Hyponatremia. Med Klin Intensivmed Notfmed. (2013) 108:53–8. doi: 10.1007/s00063-012-0120-3, 22948253

[19] Choi EY Nieves GA Jones DE. Acute stroke diagnosis. Am Fam Physician. (2022) 105:616–24. 35704804

[20] Tuano KS Seth N Chinen J. Secondary immunodeficiencies: an overview. Ann Allergy Asthma Immunol. (2021) 127:617–26. doi: 10.1016/j.anai.2021.08.413, 34481993

[21] Mahowald M Khayat M. Kidney disease: acute kidney injury. FP Essent. (2021) 509:11–9. 34643360

[22] Firth D. Bias reduction of maximum likelihood estimates. Biometrika. (1993) 80:27–38.

[23] Wen L Li M Xu T Yu X Wang L Li K. Clinical features, outcomes and prognostic factors of tuberculous meningitis in adults worldwide: systematic review and meta-analysis. J Neurol. (2019) 266:3009–21. doi: 10.1007/s00415-019-09523-6, 31485723

[24] Thao LTP Heemskerk AD Geskus RB Mai NTH Ha DTM Chau TTH . Prognostic models for 9-month mortality in tuberculous meningitis. Clin Infect Dis. (2017) 66:523–32. doi: 10.1093/cid/cix849, 29029055 PMC5850565

[25] Wang L Gu Z Chen X Yu X Meng X. Analysis of risk factors for long-term mortality in patients with stage II and III tuberculous meningitis. BMC Infect Dis. (2024) 24:656. doi: 10.1186/s12879-024-09561-0, 38956526 PMC11218231

[26] Wilkinson RJ Rohlwink U Misra UK van Crevel R Mai NTH Dooley KE . Tuberculous meningitis. Nat Rev Neurol. (2017) 13:581–98. doi: 10.1038/nrneurol.2017.120, 28884751

[27] Whitworth L Coxon J van Laarhoven A Thuong NTT Dian S Alisjahbana B . A Bayesian analysis of the association between leukotriene A4 hydrolase genotype and survival in tuberculous meningitis. eLife. (2021) 10:e61722. doi: 10.7554/eLife.61722, 33416499 PMC7793626

[28] Rohlwink UK Figaji A Wilkinson KA Horswell S Sesay AK Deffur A . Tuberculous meningitis in children is characterized by compartmentalized immune responses and neural excitotoxicity. Nat Commun. (2019) 10:3767. doi: 10.1038/s41467-019-11783-9, 31434901 PMC6704154

[29] Manyelo CM Solomons RS Walzl G Chegou NN. Tuberculous meningitis: pathogenesis, immune responses, diagnostic challenges, and the potential of biomarker-based approaches. J Clin Microbiol. (2021) 59:e01771-20. doi: 10.1128/JCM.01771-20, 33087432 PMC8106718

[30] Mpande CAM Rozot V Mosito B Musvosvi M Dintwe OB Bilek N . Immune profiling of *Mycobacterium tuberculosis*-specific T cells in recent and remote infection. EBioMedicine. (2021) 64:103233. doi: 10.1016/j.ebiom.2021.103233, 33610126 PMC7902886

[31] Doherty TM Weinberger B Didierlaurent A Lambert PH. Age-related changes in the immune system and challenges for the development of age-specific vaccines. Ann Med. (2025) 57:2477300. doi: 10.1080/07853890.2025.2477300, 40110678 PMC11926906

[32] Gong Q Sharma M Glass MC Kuan EL Chander A Singh M . Multi-omic profiling reveals age-related immune dynamics in healthy adults. Nature. (2025) 648:696–706. doi: 10.1038/s41586-025-09686-5, 41162704 PMC12711581

[33] Lourens R Singh G Arendse T Thwaites G Rohlwink U. Tuberculous meningitis across the lifespan. J Infect Dis. (2025) 231:1101–11. doi: 10.1093/infdis/jiaf181, 40197992 PMC12128070

[34] Cisneros B García-Aguirre I Unzueta J Arrieta-Cruz I González-Morales O Domínguez-Larrieta JM . Immune system modulation in aging: molecular mechanisms and therapeutic targets. Front Immunol. (2022) 13:1059173. doi: 10.3389/fimmu.2022.1059173, 36591275 PMC9797513

[35] Jang IH Niedernhofer LJ Robbins PD Camell CD. The ageing immune system as a driver of systemic ageing. Nat Rev Immunol. (2026) 26:489–506. doi: 10.1038/s41577-026-01269-3, 41688787 PMC13723950

[36] Dian S Koeken VACM Ardiansyah E Ganiem AR van Abeelen K Aguirre-Gamboa R . Inflammatory markers in the cerebrospinal fluid linked to mortality in tuberculous meningitis. Brain Commun. (2025) 7:fcaf273. doi: 10.1093/braincomms/fcaf273, 40703661 PMC12284393

[37] Hamada Y Cirillo DM Matteelli A Penn-Nicholson A Rangaka MX Ruhwald M. Tests for tuberculosis infection: landscape analysis. Eur Respir J. (2021) 58:2100167. doi: 10.1183/13993003.00167-2021, 33875495

[38] Campbell JR Pease C Daley P Pai M Menzies D. Chapter 4: diagnosis of tuberculosis infection. Can J Respir Crit Care Sleep Med. (2022) 6:49–65. doi: 10.1080/24745332.2022.2036503

[39] Pai M Denkinger CM Kik SV Rangaka MX Zwerling A Oxlade O . Gamma interferon release assays for detection of *Mycobacterium tuberculosis* infection. Clin Microbiol Rev. (2014) 27:3–20. doi: 10.1128/CMR.00034-13, 24396134 PMC3910908

