# Supplementary material for: PPD skin test positivity and in-hospital mortality across age groups in non-HIV tuberculous meningitis: a retrospective cohort study
Source: Front Neurol. 2026 Aug 14;17:1820517. doi: 10.3389/fneur.2026.1820517 (PMC13521825; doi:10.3389/fneur.2026.1820517)
Supplement: Supplementary file 1 [file Table_1.DOCX]

**Supplementary figure and tables**

**Supplementary Figure S1. Decision-curve analysis for the full-cohort mortality model with and without PPD.** Net benefit is shown across threshold probabilities from 0.05 to 0.30; treat-all and treat-none strategies are included for reference.

Supplementary Table S1. Clinical characteristics by PPD status in children

| **Variable** | **PPD negative (n=186)** | **PPD positive (n=25)** | **P value** |
| --- | --- | --- | --- |
| Age, years | 8.0 (3.0, 15.0) | 11.0 (3.0, 16.0) | 0.301 |
| Female | 94/186 (50.5%) | 17/25 (68.0%) | 0.137 |
| Immunodeficiency | 18/186 (9.7%) | 3/25 (12.0%) | 0.724 |
| Hydrocephalus | 26/186 (14.0%) | 1/25 (4.0%) | 0.212 |
| Cerebral infarction | 14/186 (7.5%) | 2/25 (8.0%) | 1.000 |
| Intracranial pressure, cmH₂O | 15.0 (15.0, 19.0) | 16.0 (15.0, 21.0) | 0.232 |
| CSF protein, g/L | 0.90 (0.60, 0.90) | 0.90 (0.42, 1.28) | 0.803 |
| In-hospital death | 13/186 (7.0%) | 9/25 (36.0%) | <0.001 |

**Supplementary Table S2. TBM diagnostic category by PPD status**

| **Population** | **TBM category** | **PPD negative** | **PPD positive** | **Fisher P** |
| --- | --- | --- | --- | --- |
| Overall | Definite | 508/1443 (35.2%) | 45/131 (34.4%) | 0.973 |
|  | Probable | 872/1443 (60.4%) | 81/131 (61.8%) |  |
|  | Possible | 63/1443 (4.4%) | 5/131 (3.8%) |  |
| Children | Definite | 99/186 (53.2%) | 13/25 (52.0%) | 1.000 |
|  | Probable | 84/186 (45.2%) | 12/25 (48.0%) |  |
|  | Possible | 3/186 (1.6%) | 0/25 (0.0%) |  |
| Adults | Definite | 409/1257 (32.5%) | 32/106 (30.2%) | 0.876 |
|  | Probable | 788/1257 (62.7%) | 69/106 (65.1%) |  |
|  | Possible | 60/1257 (4.8%) | 5/106 (4.7%) |  |

**Supplementary Table S3. Pediatric age, immunodeficiency, and PPD event counts by outcome**

| **Measure** | **Value** | **P value** |
| --- | --- | --- |
| Age among pediatric survivors, years | 8.0 (3.0, 15.0) | 0.953 |
| Age among pediatric deaths, years | 10.0 (2.2, 14.8) | 0.953 |
| Immunodeficiency among pediatric survivors | 18/189 (9.5%) | 0.466 |
| Immunodeficiency among pediatric deaths | 3/22 (13.6%) | 0.466 |
| PPD positive among pediatric survivors | 16/189 (8.5%) | <0.001 |
| PPD positive among pediatric deaths | 9/22 (40.9%) | <0.001 |

Supplementary Table S4. Model discrimination and calibration for fixed clinical models with PPD; the explicitly labeled row shows the model without PPD. The pediatric row corresponds to the primary pediatric model adjusted for age and diagnostic category.

| **Population** | **n** | **Deaths** | **AUC** | **Optimism-corrected AUC** | **Brier score** | **Calibration intercept** | **Calibration slope** |
| --- | --- | --- | --- | --- | --- | --- | --- |
| Full cohort | 1572 | 187 | 0.673 | 0.647 | 0.0994 | -0.028 | 1.009 |
| Full cohort (without PPD) | 1572 | 187 | 0.671 | 0.651 | 0.0994 | -0.025 | 1.008 |
| Children | 211 | 22 | 0.684 | 0.639 | 0.0842 | -0.067 | 1.023 |
| Adults | 1361 | 165 | 0.669 | 0.642 | 0.1010 | -0.032 | 1.014 |

Supplementary Table S5. Decision-curve net benefit in the full cohort

| **Threshold probability** | **Model with PPD** | **Model without PPD** | **Treat all** | **Treat none** |
| --- | --- | --- | --- | --- |
| 0.05 | 0.0725 | 0.0726 | 0.0726 | 0.0000 |
| 0.10 | 0.0375 | 0.0365 | 0.0211 | 0.0000 |
| 0.15 | 0.0210 | 0.0212 | -0.0365 | 0.0000 |
| 0.20 | 0.0162 | 0.0161 | -0.1013 | 0.0000 |
| 0.30 | 0.0035 | 0.0025 | -0.2586 | 0.0000 |

Supplementary Table S6. Entropy-balancing weighted outcome models for PPD positivity and in-hospital mortality

| **Analysis** | **n** | **Deaths** | **OR (95% CI)** | **P value** | **Entropy-balancing weight range** |
| --- | --- | --- | --- | --- | --- |
| Untrimmed | 1572 | 187 | 1.06 (0.59-1.90) | 0.846 | 0.398-2.479 |
| Weights truncated at 1st/99th percentiles | 1572 | 187 | 1.00 (0.58-1.74) | 0.991 | 0.648-1.428 |
